# Finite element analysis of different locking plate fixation methods for the treatment of ulnar head fracture

**DOI:** 10.1186/s13018-021-02334-4

**Published:** 2021-03-15

**Authors:** Yue Zhang, Qin Shao, Chensong Yang, Changqing Ai, Di Zhou, Yang Yu, Guixin Sun

**Affiliations:** 1 Department of Traumatic Surgery, Shanghai East Hospital, Tongji University School of Medicine, No 150, Ji Mo Road, Shanghai, 200120 China; 2 Department of Radiology, Shanghai East Hospital, Tongji University School of Medicine, Shanghai, 200120 China; 3 Walkman biomaterial CO., LTD, Tianjin, 301609 China

**Keywords:** Finite element method, Ulnar head fracture, Locking plate fixation, Distal radius and ulnar joint, Internal fixation

## Abstract

**Background:**

Ulnar head fractures are increasingly higher with the growing proportion of the elderly people. Failure to achieve a stable anatomic reduction of ulna head fracture may lead to a distal radioulnar joint (DRUJ) dysfunction and nonunion of the distal radius. Due to the lack of the postoperative reporting outcomes and the biomechanical studies, it has not been well established about the optimal management of the comminuted distal ulna head fracture. Hence, the purpose of this study is to use finite element analysis to explain the advantages and disadvantages of ulnar-side locking plate fixation compared with dorsal-side locking plate fixation and its screw arrangement in the treatment of ulnar head fractures.

**Methods:**

FE models of the ulnar head fracture and the models of ulnar-side locking plate and dorsal-side plate with two or three distal screws was constructed. In order to simulate forces acting on the ulnar and the osteosynthesis material during daily-life activity in subjects who underwent reconstructive surgery, we applied three loading conditions to each model, viz. 20 N axial compression, 50 N axial compression, 1 N∙m torsion moment, 1 N∙m lateral bending moments, and 1 N∙m extension bending moments. Under these conditions, values of the von Mises stress (VMS) distribution of the implant, peak VMS, the relative displacement of the head and shaft fragments between the fracture ends and the displacement and its direction of the models were investigated.

**Results:**

The stress values of ulnar-side plates were lower than those of dorsal-side plates. And the ulnar-plate fixation system also has smaller maximum displacement and relative displacement. When adding a screw in the middle hole of the ulnar head, the values of model displacement and the peak stress in fixation system are lower, but it may evidently concentrate the stress on the middle screw.

**Conclusions:**

In conclusion, our study indicated that ulnar-side locking plates resulted in a lower stress distribution in the plate and better stability than dorsal-side locking plates for ulnar head fracture fixation. Adding an additional screw to the ulnar head could increase the stability of the fixation system and provide an anti-torsion function. This study requires clinical confirmation of its practicality in the treatment of ulnar head fractures. This study requires clinical confirmation as to its practicality in the treatment of ulnar head fracture.

## Background

The wrist joint is one of the main joints of the human body and has high activity frequency. Previous studies have suggested that the stability of the distal radioulnar joint (DRUJ) greatly affects the function of the wrist joint, not only for forearm rotation, but also for load and force transmission [1, 2]. If a DRUJ fracture is not treated in time, it often leads to posttraumatic chronic pain and limited wrist joint activity, which greatly inconveniences the work and daily life of patients. Lack of understanding of the details of anatomy and biomechanics at the distal end of the ulna resulted in 75 years of simple resection of the distal ulna as treatment for most disabling pathologies in this part of the distal forear m[3]. In the past 25 years, one of the most exciting areas in hand surgery has been the study of anatomy, biomechanics, and pathophysiology at the distal end of the ulna [4]. Ulnar head fracture may be seen in up to 6% of patients with unstable fractures of the distal radius [5], and this rate is ever increasing due to the growing proportion of elderly people [6]. Metaphyseal distal radial fractures associated with distal ulnar shaft fractures represent an unstable injury pattern, which may cause nonunion of the distal radius [7]. In addition, researchers suspected that a significant joint reaction force can develop between the sigmoid notch of the radius and the rotationally fixed ulnar seat [8]. Thus, ulnar head fracture may also decrease forearm rotation [9]. Failure to achieve stable anatomic reduction of ulnar head fractures leads to the loss of ulnar variance and the distal ulna nonunion. Thus, may cause DRUJ dysfunction, ulnar-side wrist pain, and posttraumatic arthrosis [7, 10–12].

Due to the lack of the postoperative outcome reports and the biomechanical studies [13], the optimal management of comminuted distal ulna articular head fractures has not been well established. Ring et al. [14] reported that condylar blade plate fixation could achieve healing with good alignment, satisfactory function, and an acceptable rate of secondary surgery. David et al. [15] revealed the benefits of the application of a locked plate, which included the locked or fixed angle support, the ability to insert variable lengths of locked pegs, and a low-profile design. But dorsal locking plates may cause soft tissue complications [16]. Recently, a distal ulna hook plate has been introduced for the treatment of distal ulna fractures; however, the limitation of the vertical arrangement of distal screws may result in instability of the construct [17, 18]. In the present study, we found that ulnar-side micro-locking plates could achieve good outcomes. However, the distal radius fracture combined with ulnar head fracture is not a common clinical case, and the application of ulnar-side locking plates has never been reported before. Since the number of cases is relatively small, postoperative research is hard to perform. Through technological advancements, computer modeling has become more accurate [19]. Finite element analysis provides a convenient and accurate research method for doctors. This approach can stimulate the actual force and stress of the bone and joints [20]. The obtained results showed the potential application of finite element analysis in a wide range of numerical studies [21]. Therefore, such studies are not limited to the number of specific cases. Throughout the research history of distal ulna fractures, it has become indispensable to apply finite element analysis to evaluate the mechanical properties of implants.

Hence, the purpose of this study is to use finite element analysis to explain the advantages and disadvantages of the ulnar-side locking plate fixation, compared with the dorsal-side locking plate fixation, and its screw arrangement in the treatment of the ulnar head fractures.

## Methods

### Establishment of the finite element models

**Fig. 1:**
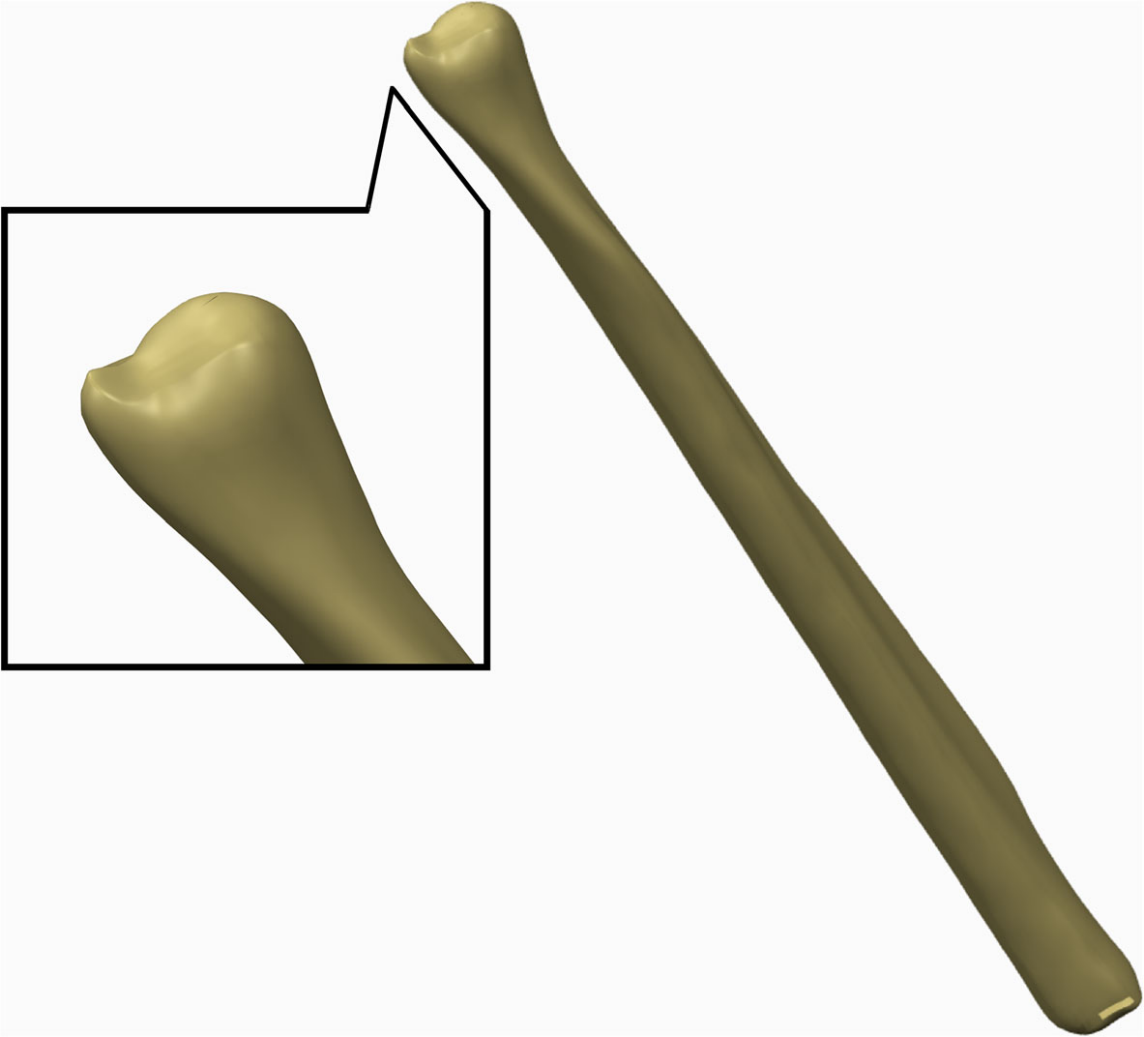
3D model of the right ulna built by Geomagic 12

A 45-year-old healthy female volunteer without a history of wrist and systemic diseases was recruited for the study. A Canon Aquilion ONE ViSION Edition CT scanner was used to perform a high-resolution CT scan of her right forearm. The scanning layer thickness was 0.5 mm. The CT scan was stored as a DICOM file in Mimics 19.0. The reconstruction slice thickness was 0.5 mm. A 3D model of the right forearm was obtained based on the gray value of the tissue and segmentation of the region and was exported as an IGES file and then incorporated into Geomagic 12 for smoothing, meshing, and fitting surface processing (Fig. 1).

**Fig. 2:**
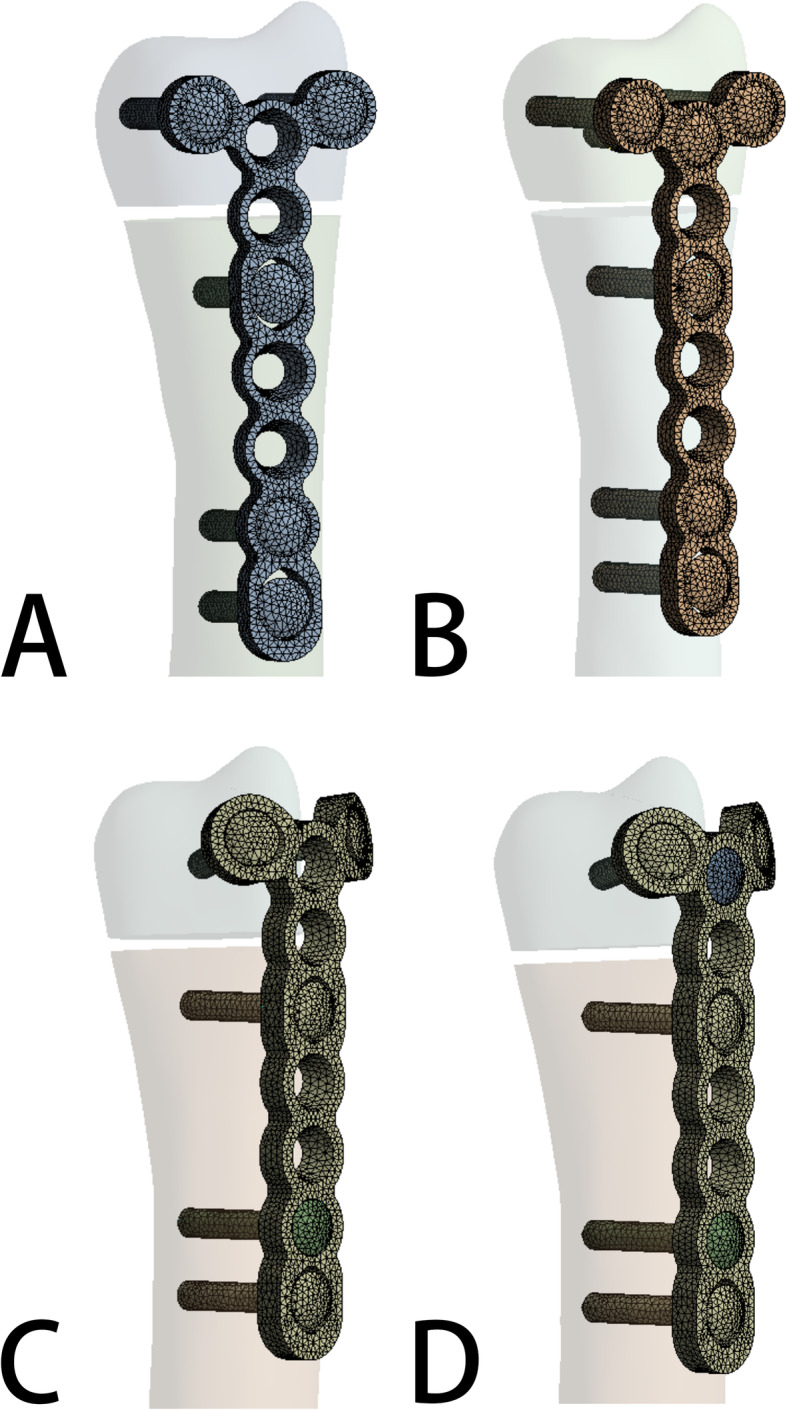
**a** Model of dorsal side locking plate with two distal screws. **b** Model of dorsal side locking plate with three distal screws. **c** Model of ulnar side locking plate with two distal screws. **d** Model of ulnar side locking plate with three distal screws

Then, the model was incorporated into the Creo Parametric 2.0. In this study, we used an OsteoMED 2.0 HPS Y-plate system. Thus, cannulated screws with a diameter of 2.0 mm and Y-steel plates were fabricated using Creo Parametric 2.0. A model of the ulnar head was established and stabilized with an ulnar-side plate and a dorsal-side plate respectively according to a practical surgical method with no interfragmentary gap (Fig. 2). The implant material was modeled as titanium alloy Ti6Al4V with the following material constants: elastic modulus *E*=110 Gpa and Poisson’s ratio *μ* = 0.33.

**Fig. 3:**
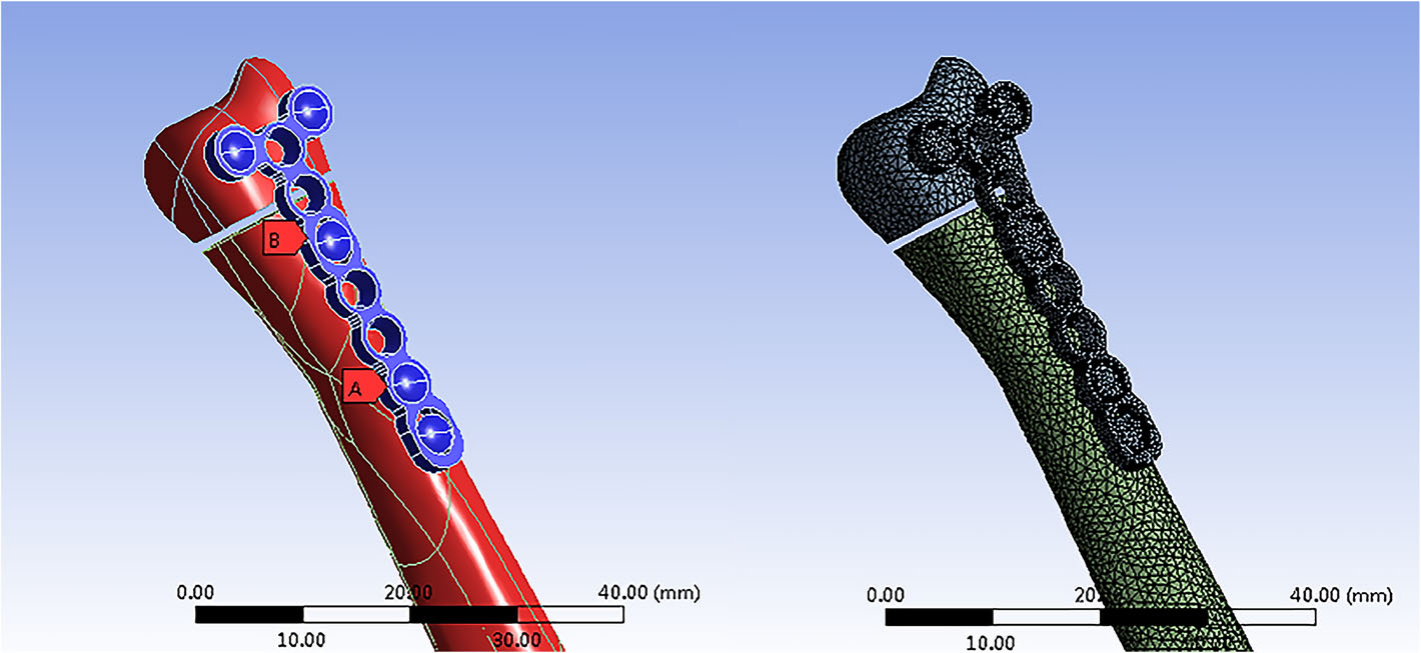
Stablishing the internal fixation models of ulnar head fracture and importing the models into Abaqus software for meshing

Subsequently, the models were incorporated into ANSYS Workbench 15.0 for meshing, and the fracture line of the ulnar head fracture was cut as described by Paksima [13]. When there are more than two geometric models, the relative relationship between the models should be set according to the actual situation, so we set the contact setting to a bonded relation in this report (Fig. 3).

The bone was defined with linear elastic material properties using a Young’s modulus of 17 GPa for the cortical bone and 1.5 GPa for the cancellous bone. The Poisson’s ratio for both the cortical and cancellous bones was 0.3 [22]. A three-dimensional model of the cortical bone and cancellous bone was developed by Boolean operations, and the proximal femoral bone model was built for reassembly.

### Loading force settings

**Fig. 4:**
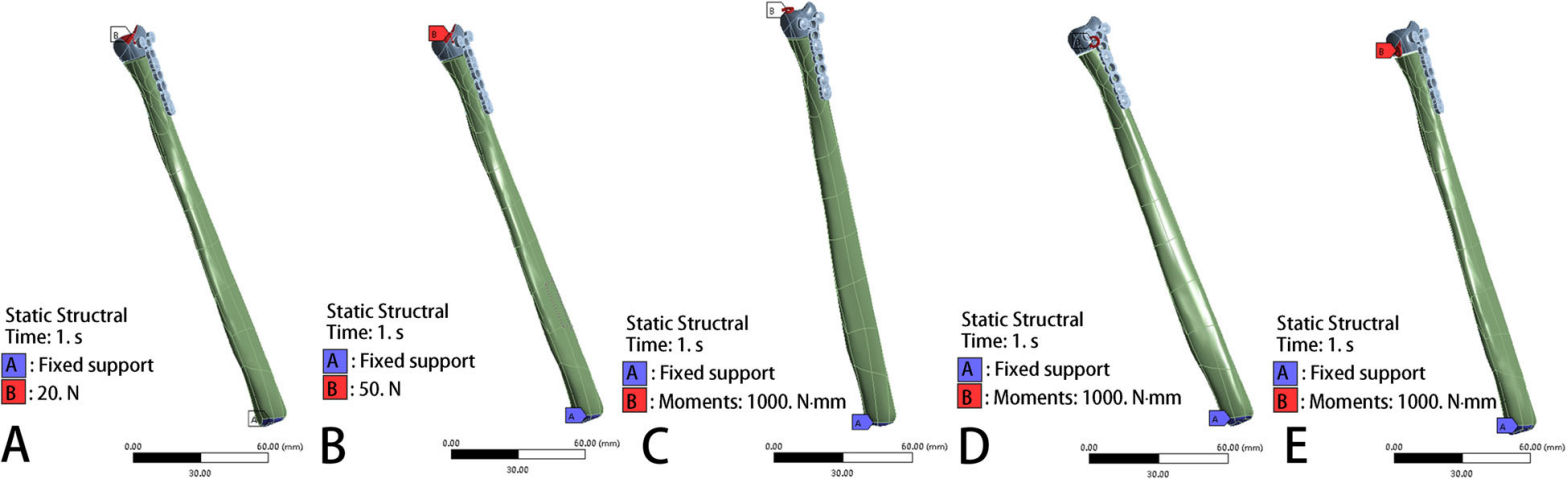
Loading force settings: **a** 20N Axial compression. **b** 50N Axial compression. **c** 1 N∙m Torsion moments. **d** 1 N∙m lateral bending moments. **e** 1 N∙m extension bending moments

In vivo loading conditions in the human DRUJ have not been completely determined. Bernal et al. [23] found that the mean grip force was 18.6 N when performing a daily life activity by measuring different subjects through wearable capacitive pressure sensors in the fingers. Putnam et al. [24] reported that each 10 N of grip force would transmit 26 N of force through the distal ulna metaphysis in the wrist neutral position. If the wrist was no longer in extension and ulnar deviation owing to the variation in hand position during a power grip, the amount of force through the distal ulna metaphysis would decrease. Shaaban et al. [25] reported that the loading of the hand could create an anterior bending force in the distal ulna in half of the forearm and a posterior bending force in the remaining half. Gordon et al. found that a positive bending moment about the medial-lateral axis results from a posteriorly directed joint reaction force, whereas a positive bending moment about the anterior-posterior axis results from a medially directed joint reaction force [3]. Thus, to simulate forces acting on the ulnar and the osteosynthesis material during daily life activity in subjects who underwent reconstructive surgery, we applied the following loading conditions to each model: 20 N axial compression, 50 N axial compression, 1 N∙m torsion moment, 1 N∙m lateral bending moment, and 1 N∙m extension bending moment (Fig. 4) [26–28].

### Evaluation criteria of the system

First, the maximum displacement and its direction of the model were measured. Second, the von Mises stress (VMS) distribution and peak VMS of both the fixation plates and the internal fixation system were observed for four models [29]. Then, the relative displacement of the head and shaft fragments between the fracture ends, which was used to evaluate the support effects, was calculated by measuring the displacement in each direction of the XYZ axis. Finally, the von Mises stress (VMS) distribution and the displacement of the four different models were plotted as a nephogram [30–32]. These parameters were used to capture the mechanical factors involved in the fixation stability and fracture healing [33].

## Results

### The von Mises stress (VMS) distribution

**Fig. 5:**
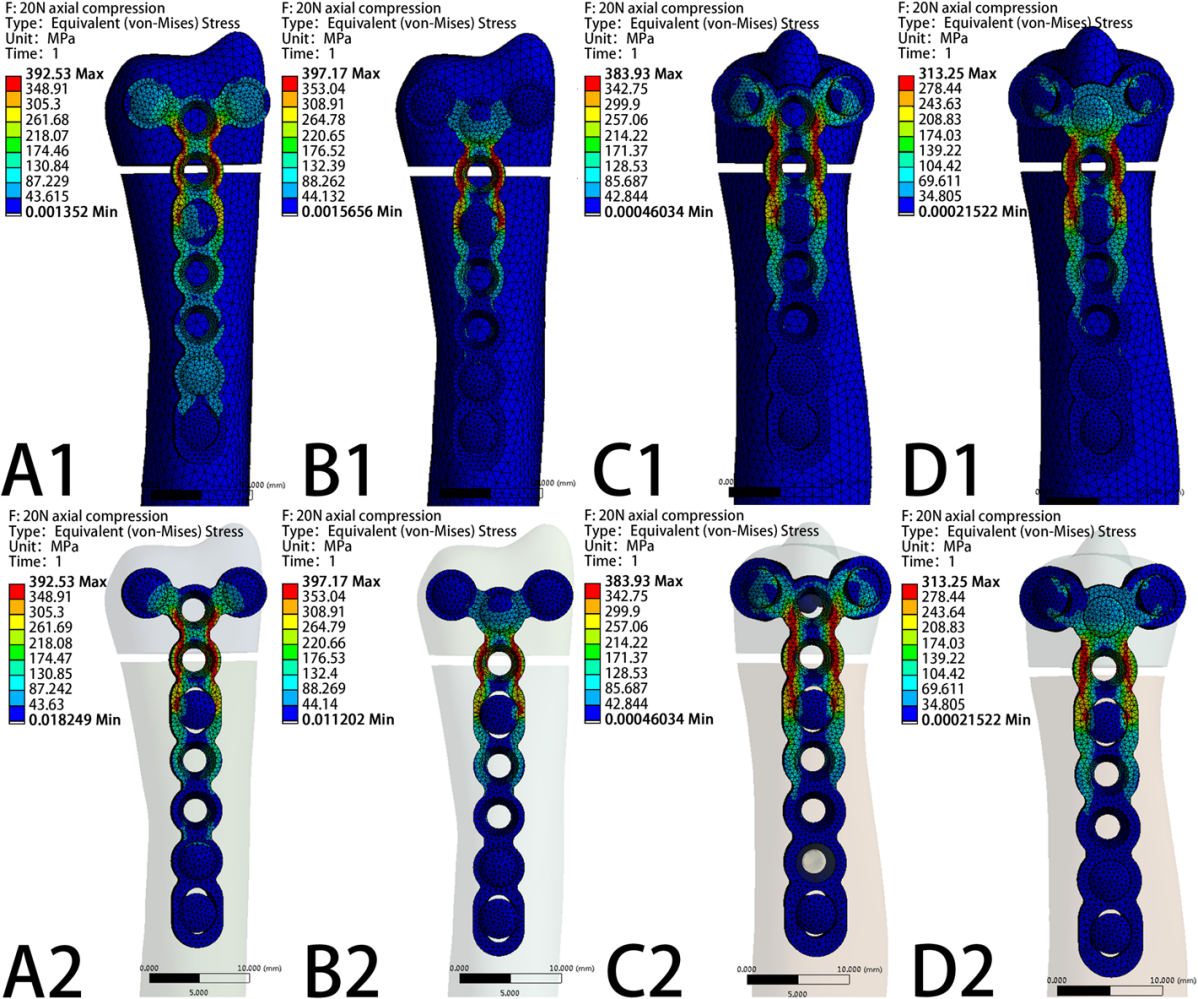
Von Mises stress distribution of models and fixation plates in four different fixation systems under 20N axial compression. **a** Model of dorsal side locking plate with two distal screws. **b** Model of dorsal side locking plate with three distal screws. **c** Model of ulnar side locking plate with two distal screws. **d** Model of ulnar side locking plate with three distal screws

**Fig. 6:**
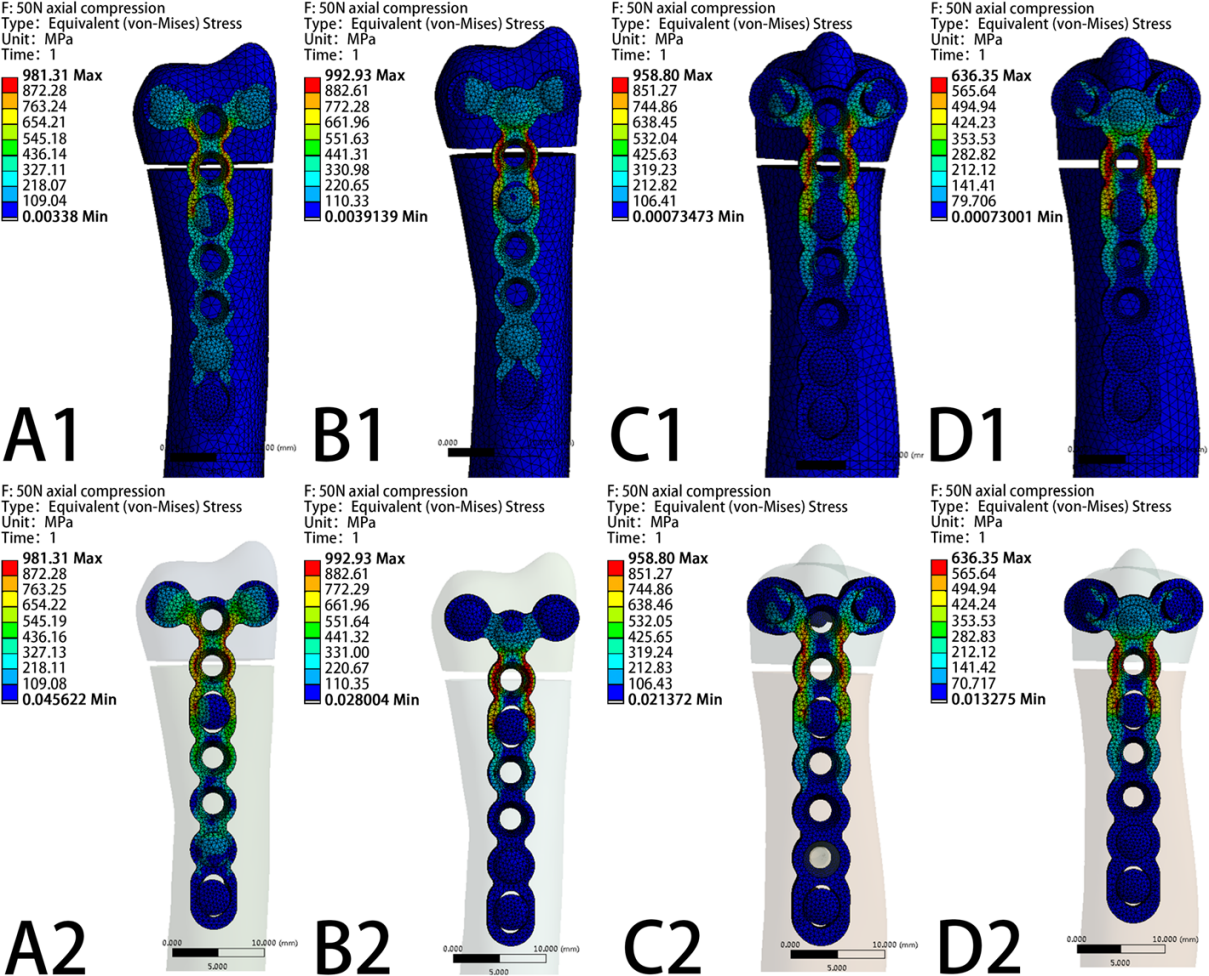
Von Mises stress distribution of models and fixation plates in four different fixation systems under 50N axial compression. **a** Model of dorsal side locking plate with two distal screws. **b** Model of dorsal side locking plate with three distal screws. **c** Model of ulnar side locking plate with two distal screws. **d** Model of ulnar side locking plate with three distal screws

**Fig. 7:**
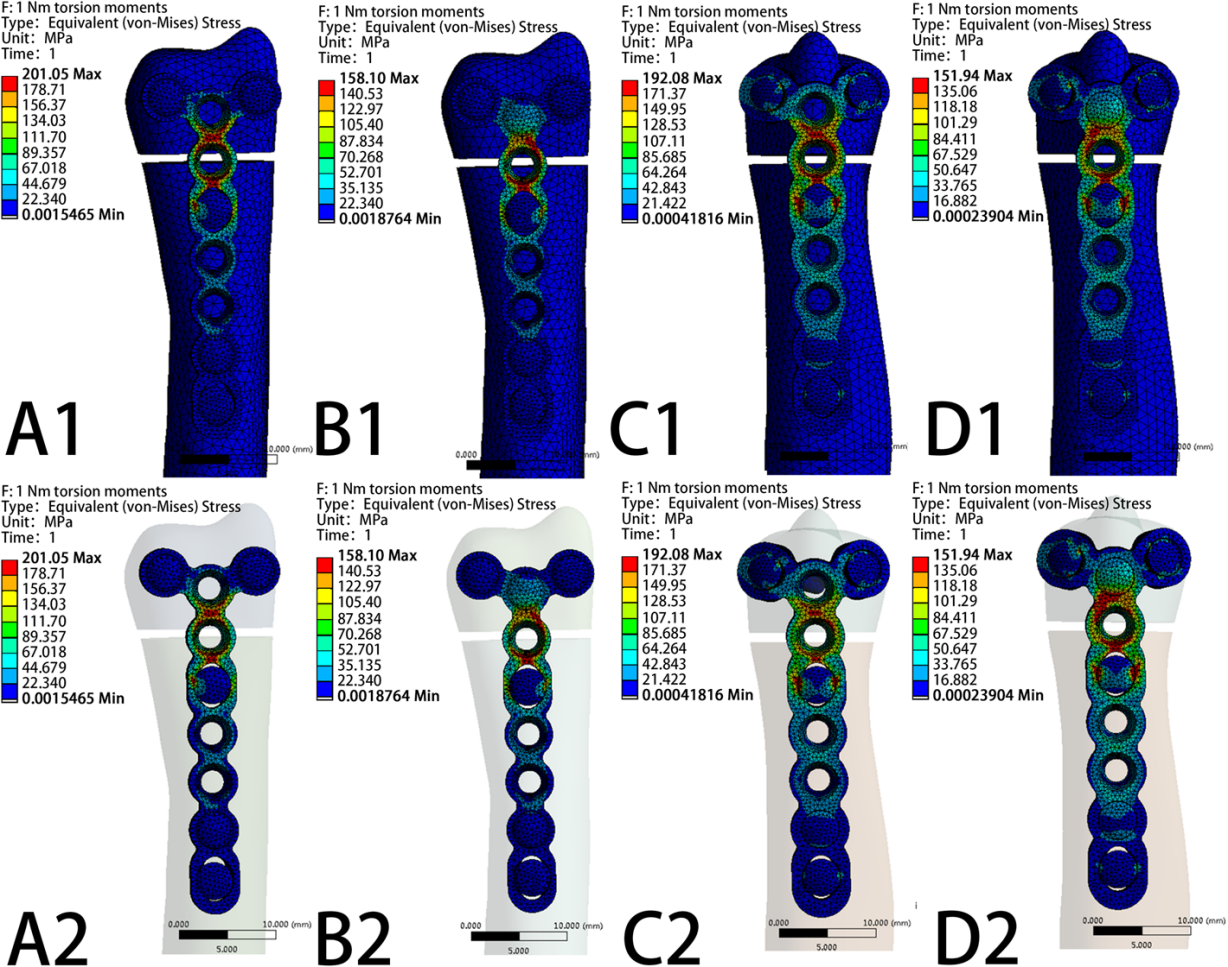
Von Mises stress distribution of models and fixation plates in four different fixation systems under 1 N∙m Torsion moments. **a** Model of dorsal side locking plate with two distal screws. **b** Model of dorsal side locking plate with three distal screws. **c** Model of ulnar side locking plate with two distal screws. **d** Model of ulnar side locking plate with three distal screws

**Fig. 8:**
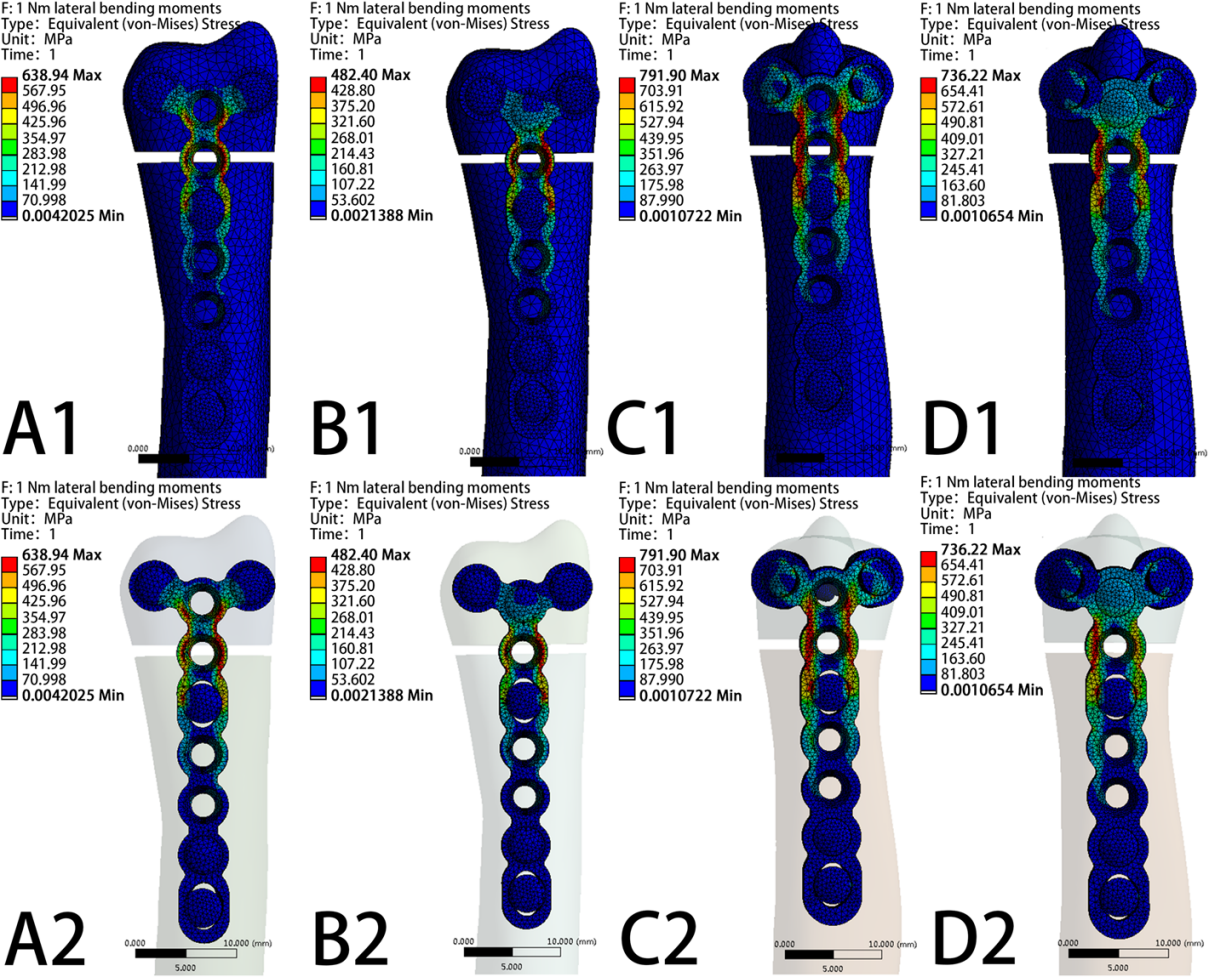
Von Mises stress distribution of models and fixation plates in four different fixation systems under 1 N∙m lateral bending moments. **a** Model of dorsal side locking plate with two distal screws. **b** Model of dorsal side locking plate with three distal screws. **c** Model of ulnar side locking plate with two distal screws. D Model of ulnar side locking plate with three distal screws

**Fig. 9:**
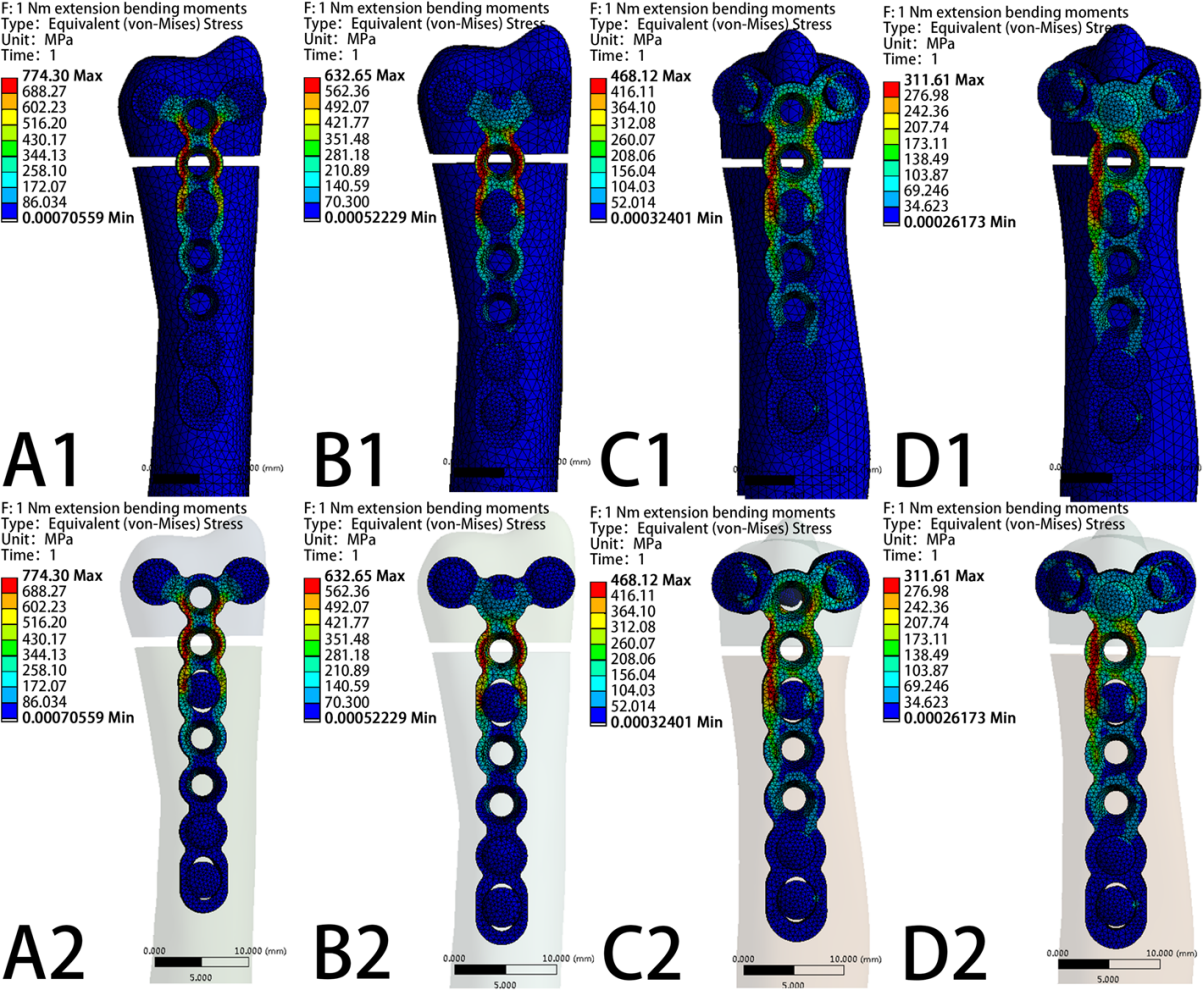
Von Mises stress distribution of models and fixation plates in four different fixation systems under 1 N∙m extension bending moments. **a** Model of dorsal side locking plate with two distal screws. **b** Model of dorsal side locking plate with three distal screws. **c** Model of ulnar side locking plate with two distal screws. **d** Model of ulnar side locking plate with three distal screws

**Fig. 10:**
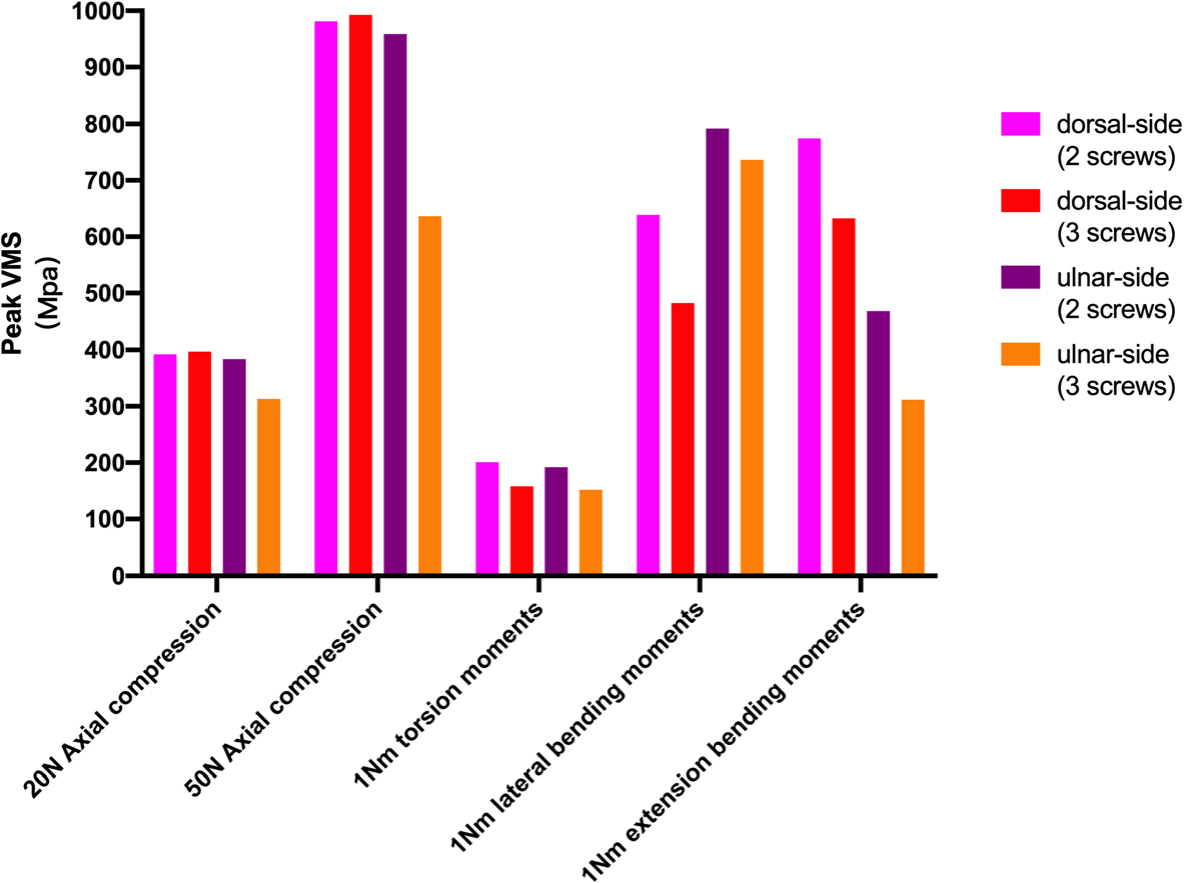
The peak Von Mises stress distribution of four different fixation systems under 5 loading settings

The VMS patterns of the five loading settings—20 N axial compression, 50 N axial compression, 1 N∙m torsion moment, 1 N∙m lateral bending moment, and 1 N∙m extension bending moments—in the four fixation systems are shown in Fig. 5, Fig. 6, Fig. 7, Fig. 8, and Fig. 9, respectively. The stress values of the ulnar-side plate were lower than those of the dorsal-side plate. In 5 loading settings, obvious stress concentrations were found near the fracture line in the 4 models. The maximum von Mises stress on the fixation plate is recorded in Table 1 and Fig. 10. Thus, the maximum von Mises peak stress of the ulnar-side fixation plate was lower, which indicated that the ulnar-side fixation plate could smoothly transfer the load to the proximal cortical bone. The peak stress in the fixation system under 3 rotating moments were apparently reduced by adding a screw in the middle hole of the ulnar head. Although the peak stress decreased only in ulnar-side plate fixation under axial compression, it may evidently concentrate the stress on the middle screw in four fixation systems.

**Table 1:** The maximum Von Mises peak stresses on fixation plate

	Dorsal-side plate (2 screws)	Dorsal-side plate (3 screws)	Ulnar-side plate (2 screws)	Ulnar-side plate (3 screws)
20N axial compression	392.53MPa	397.17Mpa	383.90Mpa	313.25Mpa
50N axial compression	981.31Mpa	992.93Mpa	958.80Mpa	636.35Mpa
1 N∙m torsion moments	201.05Mpa	158.10Mpa	192.08Mpa	151.94Mpa
1 N∙m lateral bending moments	638.94 MPa	482.40MPa	791.90 MPa	736.22 MPa
1 N∙m extension bending moments	774.30 MPa	632.65 MPa	468.12 MPa	311.61 MPa

### Fracture displacement changes

**Fig. 11:**
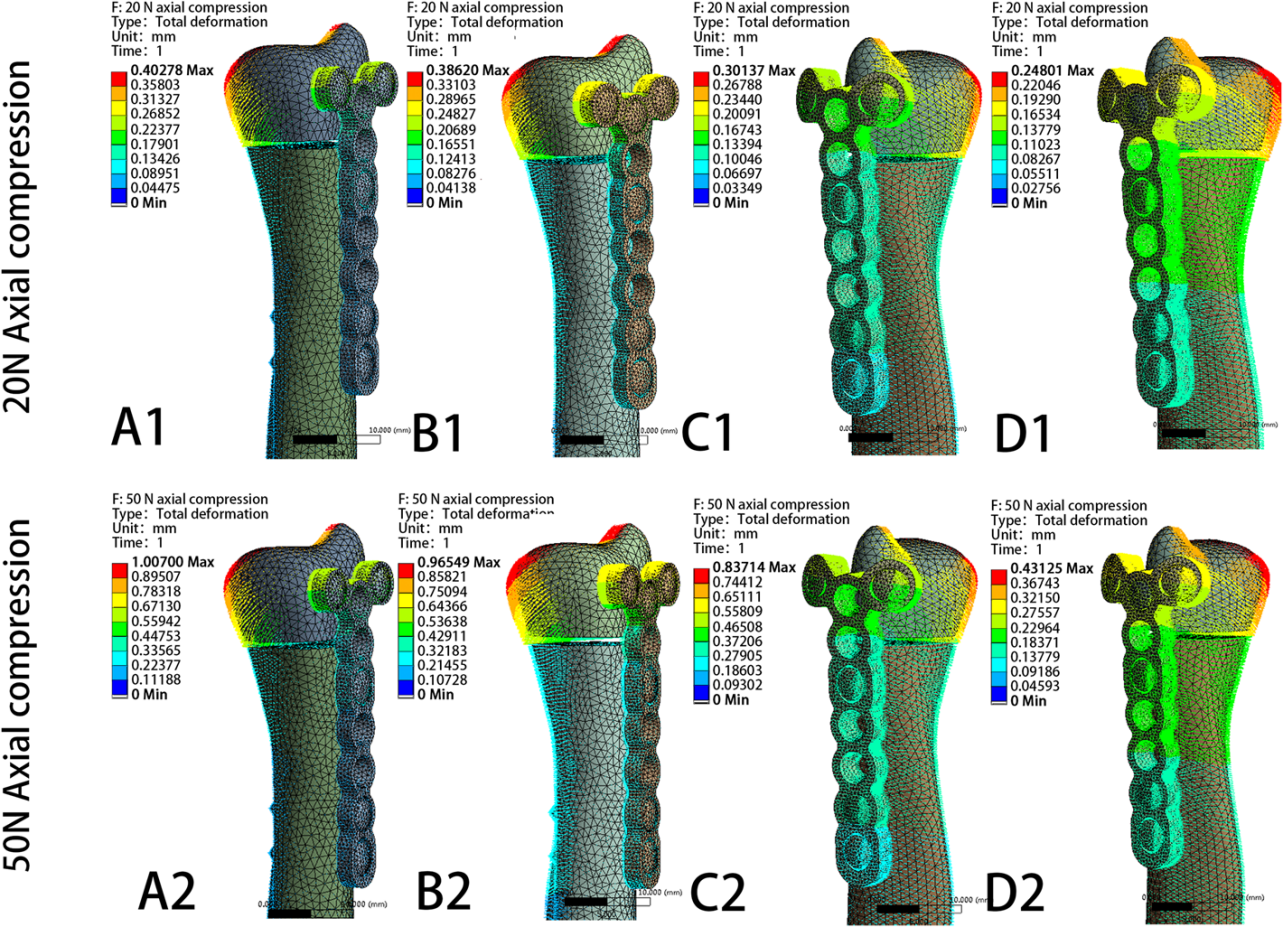
The model displacement patterns with direction of four different fixation plates under 20N and 50N axial compression loading settings. **a** Model of dorsal side locking plate with two distal screws. **b** Model of dorsal side locking plate with three distal screws. **c** Model of ulnar side locking plate with two distal screws. **d** Model of ulnar side locking plate with three distal screws

**Fig. 12:**
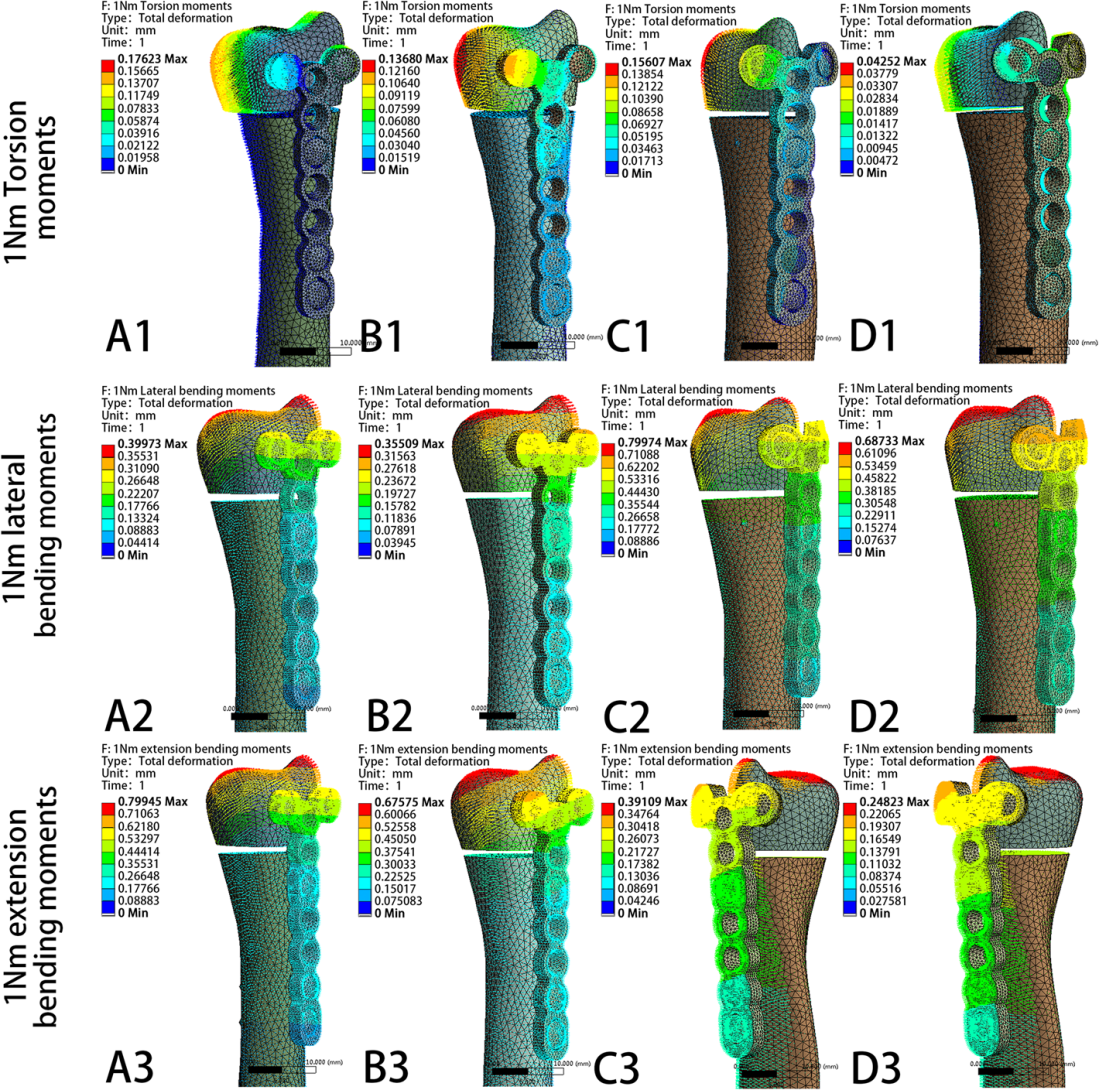
The model displacement patterns with direction of four different fixation plates under 1 N∙m torsion moments, 1 N∙m lateral bending moments, and 1 N∙m extension bending moments loading settings. **a** Model of dorsal side locking plate with two distal screws. **b** Model of dorsal side locking plate with three distal screws. **c** Model of ulnar side locking plate with two distal screws. **d** Model of ulnar side locking plate with three distal screws

**Fig. 13:**
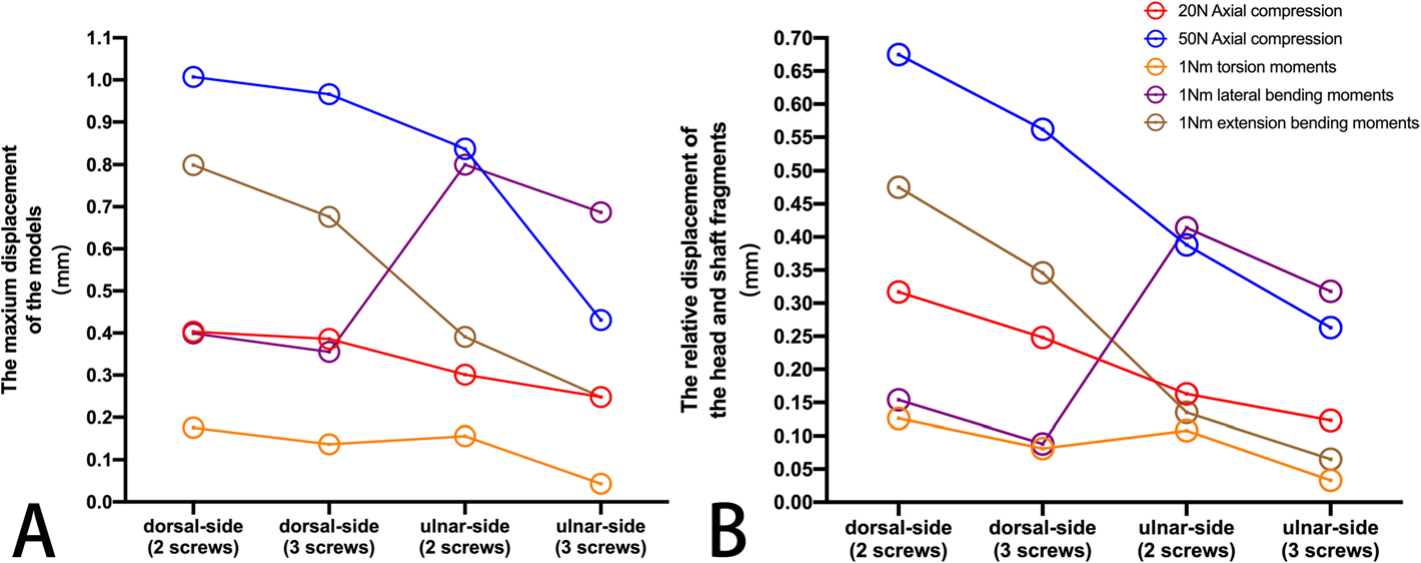
**a** The maximum displacement of 4 models under 5 loading settings. **b** The relative displacement of the head and shaft fragments in 4 models under 5 loading settings

The model displacement patterns in the four fixation systems under the two axial compression and three rotating loading settings are shown in Fig. 11 and Fig. 12, respectively. The maximum displacement and the relative displacement of the 4 models are shown in Table 2, Table 3, and Fig. 13. It is clear that the ulnar plate fixation system has smaller maximum displacement and relative displacement, which reflects not only better system stability but also less friction and movement between the head and shaft fragments.

**Table 2:** The maximum displacement of the 4 models

	Dorsal-side plate (2 screws)	Dorsal-side plate (3 screws)	Ulnar-side plate (2 screws)	Ulnar-side plate (3 screws)
20N axial compression	0.403 mm	0.386 mm	0.301 mm	0.248 mm
50N axial compression	1.007 mm	0.966 mm	0.837 mm	0.431 mm
1 N∙m torsion moments	0.176 mm	0.137 mm	0.156 mm	0.043 mm
1 N∙m lateral bending moments	0.400 mm	0.355 mm	0.800 mm	0.687 mm
1 N∙m extension bending moments	0.799 mm	0.676 mm	0.391 mm	0.248 mm

**Table 3:** The relative displacement of the head and shaft fragments in 4 models

	Dorsal-side plate (2 screws)	Dorsal-side plate (3 screws)	Ulnar-side plate (2 screws)	Ulnar-side plate (3 screws)
20N axial compression	0.317 mm	0.248 mm	0.163 mm	0.123 mm
50N axial compression	0.675 mm	0.562 mm	0.388 mm	0.263 mm
1 N∙m torsion moments	0.126 mm	0.081 mm	0.108 mm	0.033 mm
1 N∙m lateral bending moments	0.154 mm	0.088 mm	0.414 mm	0.318 mm
1 N∙m extension bending moments	0.475 mm	0.346 mm	0.135 mm	0.065 mm

## Discussion

The static stability of the DRUJ is achieved by the bony congruity between the sigmoid notch of the radius and the ulnar head and by the ligaments that hold the joint together [34]. Part of the ligaments constitutes the main stabilizer of the DRUJ, which runs from the fovea of the ulnar head to the dorsal and palmar edges of the sigmoid notch on the distal radius [35–37]. The distal interosseous membrane (DIOM) of the forearm acts as a secondary soft tissue stabilizer of the DRUJ. The DIOM originates from the distal ulna 54 mm (on average) proximal to the ulnar head and runs distally to insert on the dorsal inferior rim of the sigmoid notch of the radius, which is at the end of the central band of the interosseous membrane [38–40]. Therefore, when the ulnar head breaks, the ligament will lose its stable attachment point, resulting in the instability of the DRUJ. Distal ulnar metaphyseal fracture can be deemed as a fracture ranging from the ulnar neck to within 5 cm of the distal dome of the ulnar head and its high incidence of it is related to osteoporosis [41]. Since 2000, with the development of internal fixation technology and the increasing population of elderly people, the requirements for the recovery of wrist joint function have gradually increased. An increasing number of surgeons choose open reduction and internal fixation to treat unstable distal ulnar fractures [42, 43]. Palmer and Werner [44] showed that up to 42% of force passes through the ulna, in which the axial force passing down the ulnar head fracture end was closer to 20% [44]. The above studies indicated that the loss of the ulna head would disrupt the biomechanics and load-bearing capacity of the DRUJ. Therefore, the demand for internal fixation treatment has increased owing to the biomechanical characteristics of ulnar head fractures.

However, the number of reported cases and literature studies is rather sparse, which is mostly limited by the low incidence, merely 5–6%, of distal radius fractures accompanied by a distal ulnar metaphyseal fractur e[5].. At present, the treatment of distal ulnar head fractures remains controversial. It is challenging to perform an internal fixation of distal ulnar metaphyseal fractures because the distal fracture fragment is small, comminuted, osteoporotic, and covered with an articular surface over a 270° arc, and surgical exposure of the distal ulna for hardware placement introduces the possibility of damaging the dorsal sensory branch of the ulnar nerve [45]. The most widely used fixation methods are dorsal micro-locking plates and anatomical hook plates, but their merits, drawbacks, and mechanical properties remain unclear. Although the hook plate conforms to the ulnar anatomical structure of the distal ulna, there are few screw holes in the head that are arranged vertically, and the screw placement is limited during the operation. On the other hand, the locking plate has more screw holes and the characteristics of pre-bending. Considering that the horizontal arrangement of screws has a higher anti-rotation ability, we propose a method of placing the micro-locking plate on the ulnar side. Nevertheless, limited by the number of clinical cases, retrospective studies are difficult to carry out. Therefore, a new method of analysis is urgently needed.

Currently, thanks to the latest development of finite element model generation, such as improved quality of CT imaging, segmentation algorithms, and computing power, the accuracy of finite element modeling has been greatly increased [46]. With the maturity of technology, 3D finite element analysis (FEA) can simulate the biomechanical analysis of complex orthopedic diseases and eliminate the limitation of the lack of cases. In this study, we chose to use FEA to determine whether placing an ulnar-side locking plate has better biomechanical properties than the current choice of a dorsal-side locking plate. We hope the mechanical results of this study provide experimental guidance for its application in clinical surgeries.

As shown in Table 2, Table 3, and Fig. 13, the ulnar-side locking plate models provided more stable fixation than the dorsal-side models, and the stability increased from the increased number of head screws. According to the direction of the displacement shown in Figs. 11 and 12, axial compression is more likely to produce palmar, proximal, and lateral movement. Under a torsion moment, the radial side of the ulnar head produces more radial-palmar displacement. When the lateral bending force is applied on the ulnar side, the ulnar head fragments move diagonally towards the distal side to the ulnar and dorsal sides. Proportionately, when the extension bending force is applied, the ulnar head fragments move to the ulnar and dorsal sides. Figure 5 through 9 illustrate that the stress of the four fixation systems was concentrated at the fracture line. Both the stress concentration zone and the maximum displacement were decreased in ulnar-side locking plate fixation. As shown in Table 1 and Fig. 10, under torsion, extension, and lateral bending moments, the peak VMS in the four fixation models decreased with the addition of the ulnar head screw, which evidently indicated the anti-torsion function of the ulnar head screw. Under axial loading, the peak VMS increased in the dorsal-side fixation models and concentrated at the additional middle screw, whereas it decreased in the ulnar-side fixation models. Under the same bending force action to the plate, the deformation and the peak VMS of the dorsal-side fixation models at 1 Nm extension moments was basically equal to the ulnar-side fixation models at 1 Nm lateral bending moments, while the latter is still smaller. The abovementioned results indicate that ulnar-side locking plate fixation provided better stability, resulting in a lower stress distribution in the plate and greater security of the fixation system. Because of the anatomical structure of the distal ulna, ulnar-side plate had greater distal curvature after pre-bending, which reduced the lifting displacement of the fracture end according to the gauss theorem egregium. In addition, the angle of the head screw in ulnar-side fixation provided better distal angulation stability, so that the strength of pulling out the screw was increased [47]. Thus, we considered that the ulnar-side plate fixation could generate a rigid, stable mechanism and provide a strong resistance to counter compression and torsion effects. Adding the additional screw enabled the fixation models to generate better stability but concentrated the stress on the middle screw, which will guide the design of subsequent plate improvement. This study is the first FEA comparing the mechanical efficiency of dorsal-side locking plates and ulnar-side locking plates in the fixation of ulnar head fractures. However, with the limitation that no experimental validation was conducted and no soft tissue structure was included in the models, the application of these fixation plates still requires more research.

This finite element simulation may facilitate further mechanical researches and provide guidance for the clinical treatment of the ulnar head fractures.

## Conclusions

In conclusion, our study indicated that ulnar-side locking plates resulted in a lower stress distribution in the plate and better stability than dorsal-side locking plates for ulnar head fracture fixation. Adding an additional screw to the ulnar head could increase the stability of the fixation system and provide an anti-torsion function. This study requires clinical confirmation of its practicality in the treatment of ulnar head fractures.

## Data Availability

The datasets used and/or analyzed during the current study are available from the corresponding author on reasonable request.

## Abbreviations

DRUJ: Distal radius and ulna joint
CT: Computed tomography
VMS: Von Mises stress
DIOM: Distal interosseous membrane
FEA: Finite element analysis
